# An empirical study on Resource Description Framework reification for trustworthiness in knowledge graphs

**DOI:** 10.12688/f1000research.72843.2

**Published:** 2021-11-29

**Authors:** Sini Govindapillai, Lay-Ki Soon, Su-Cheng Haw

**Affiliations:** 1 Faculty of Computing Informatics, Multimedia University, Cyberjaya, Selangor, 63100, Malaysia; 2 School of Information Technology, Monash University Malaysia, Bandar Sunway, Selangor, 47500, Malaysia

**Keywords:** Wikidata, YAGO, RDF reification, Knowledge Graph, provenance data

## Abstract

Knowledge graph (KG) publishes machine-readable representation of knowledge on the Web. Structured data in the knowledge graph is published using Resource Description Framework (RDF) where knowledge is represented as a triple (subject, predicate, object). Due to the presence of erroneous, outdated or conflicting data in the knowledge graph, the quality of facts cannot be guaranteed. Trustworthiness of facts in knowledge graph can be enhanced by the addition of metadata like the source of information, location and time of the fact occurrence. Since RDF does not support metadata for providing provenance and contextualization, an alternate method, RDF reification is employed by most of the knowledge graphs. RDF reification increases the magnitude of data as several statements are required to represent a single fact. Another limitation for applications that uses provenance data like in the medical domain and in cyber security is that not all facts in these knowledge graphs are annotated with provenance data. In this paper, we have provided an overview of prominent reification approaches together with the analysis of popular, general knowledge graphs Wikidata and YAGO4 with regard to the representation of provenance and context data. Wikidata employs qualifiers to include metadata to facts, while YAGO4 collects metadata from Wikidata qualifiers. However, facts in Wikidata and YAGO4 can be fetched without using reification to cater for applications that do not require metadata. To the best of our knowledge, this is the first paper that investigates the method and the extent of metadata covered by two prominent KGs, Wikidata and YAGO4.

## Introduction

Knowledge regarding the real-world entities in machine-readable format is furnished by Knowledge Graphs (KGs). These large-scale KGs provide both domain-dependent and domain-independent knowledge for many applications like entity linking, information retrieval and several other data mining tasks. KGs can be created by the extraction of structured knowledge from data sources like Wikipedia, collected by Artificial Intelligent (AI) projects, imported from other data sets, or by crowd-sourcing. Regardless of the methods followed, the presence of noise diminishes the quality of the KGs. ^1^

Trustworthiness can be defined as “the degree to which the information is accepted to be correct, true, real, and credible”. ^2^ Trust in the data can be increased by providing additional information like the source of information or contextual information like the time or the location in which this fact was true or any other relevant additional information pertaining to a fact. ^3^ This extra information would support the authenticity of the data, and in return, can help the machines to extract correct facts for critical applications such as in medical domain and in cyber security.

However, data in KGs are published using Resource Description Framework (RDF) ^4^ where RDF encodes facts in the form of triples. RDF statement is an ordered set of the form (s, p, o) where s and o describe the subject and object entities and p describes the relationship between these two entities.

RDF triple can be represented as:

(s, p, o) ∈ (I ∪ B) × I × (I ∪ B ∪ L)

• I is the set of Internationalized Resource Identifiers (IRIs), which identifies a unique resource on the Web
• L is the set of RDF Literals,
• B is the set of blank nodes that are anonymous and cannot be dereferenced globally.

**Figure 1.**
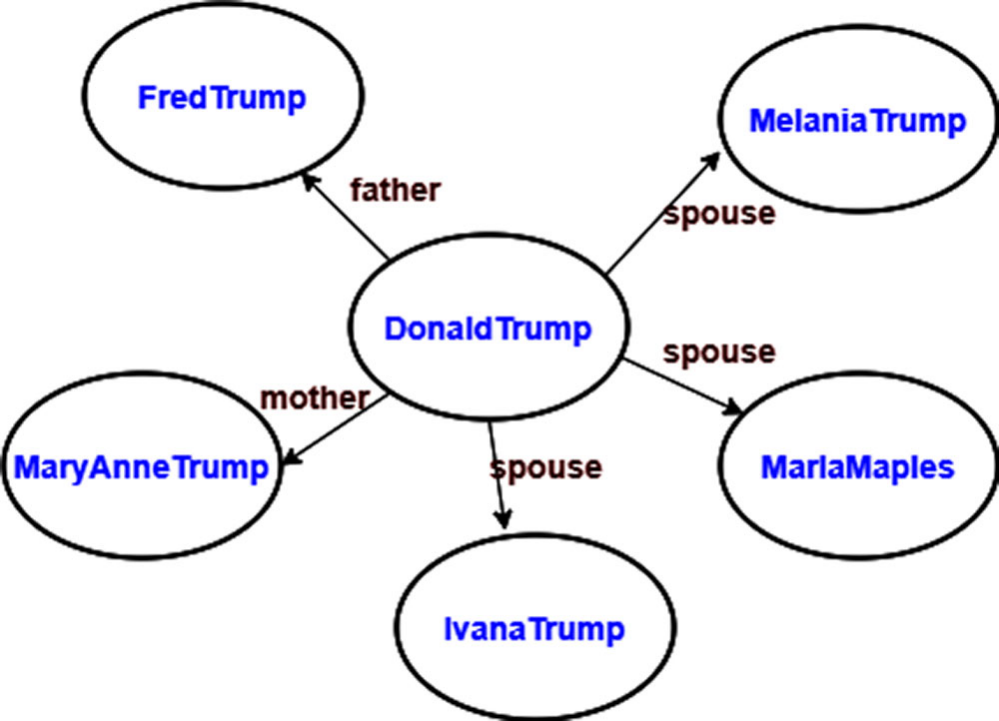
Sample RDF triples of the entity Donald Trump.

Figure 1 depicts examples of personal facts of the entity Donald Trump expressed as RDF triples. Some facts are eternal truth such as an individual’s parents, while some other facts can be true at a certain point in time. This figure shows that Donald Trump has three wives, which is partially correct. Donald Trump was married to three entities at different points in time. This example shows the importance of contextual data and the inadequacy of RDF in its expression.

However, RDF data model does not support adding metadata to triples and therefore provenance data cannot be attached. Sikos and Philip ^5^ describes different scales of provenance for datasets. In this paper, we focus on the provenance and contextualization of RDF data at the triple level. We first look at the existing approaches for integrating metadata into RDF. Next, we explore two popular KGs, namely Wikidata ^6, 7^ and YAGO ^8^ to see how and to what extent they support meta information.

### Reification approaches

Trustworthiness on the KG level is dependent on the trustworthiness of the facts at the basic level, which is the triple level. Contextual data restricts or gives meaning to a specific situation, location or time where this fact is true. Provenance data add source from which this triple is curated or imported. Therefore, we hypothesize that both contextual and provenance data adds trust to the fact that is described.

In order to encapsulate this additional information to RDF triple, RDF reification is used. In this section, we will explore some of the available methods for reifying RDF triples. These approaches include metadata of statements as triples in KGs. Additionally, some of the popular reification approaches standard reification, singleton property, n-ary relations and RDF* will be discussed.

### Standard reification

Standard reification ^4^ annotates facts using RDF built-in vocabulary and the concept of blank nodes as described in RDF Primer. In standard reification, four triples are used for representing a fact. Blank nodes of type *Statement* together with properties subject, predicate and object are used for representing the triple. Additional provenance data can then be added to these triples using the same blank node.

For example, the fact *Serena Williams is born in Saginaw* can be represented as RDF statement (SerenaWilliams, birthPlace, Saginaw). The source of the triple is given as the book *History of Tennis by Bud Collins.* Standard reification employing blank node labelled _:x is used for reification in Table 1. Provenance information is added as the last row in the table using the same blank node _:x. Any number of metadata can be added with this blank node as the subject.

**Table 1.** Standard reification.

Subject	Predicate	Object
_:x	rdf:type	rdf:Statement
_:x	rdf:subject	SerenaWilliams
_:x	rdf:predicate	birthPlace
_:x	rdf:object	Saginaw
_:x	dc:source	HistoryOfTennis

Here for representing a single fact, four triples are needed. This will magnify the size of the KG by four times. At the same time, queries for retrieving these metadata facts will become complicated. This simple method of reification does not have formal semantics to link reified and original triples, and it is not commonly used.

### Singleton property

In order to overcome issues with standard reification, s *ingleton property* ^9^ was proposed. Singleton property provided formal semantics to RDF reification and the number of triples describing fact is reduced. This reification is based on the idea that the relation between two specific entities is unique. This unique statement-level property can be an instance of the general predicate using *singletonPropertyOf* property, which is a sub-property of rdf:type. This unique predicate is used to attach additional information to the fact like contextualization or provenance information.

In the example given in Table 2, birthplace_1 is an instance of *singleton property* describing the relation birthPlace between entities Serena Williams and Saginaw. Each of these relations has a unique IRI. Any number of metadata can be added to this fact with singleton property birthplace_1 as the subject. This will introduce a large number of unique predicates in the KG. This can be problematic for indexing techniques adopted by current triplestores.

**Table 2.** Singleton property.

Subject	Predicate	Object
Serena Williams	birthplace_1	Saginaw
birthplace_1	rdf:singleton-PropertyOf	birthPlace
birthplace_1	dc:source	HistoryOfTennis

### n-ary relations

Another alternative to achieve reification is the use of the *n-ary* relations. ^6, 10^ In this model, the subject has a relationship with some qualifiers and values. Resource node is used for this, but unlike standard reification, it is attached to the original triple.

Example in Table 3 depicts *n-ary* relation of the triple (SerenaWilliams, birthPlace, Saginaw) where subject Serena Williams is related to a resource node, which cannot be dereferenced on the Web. This resource birthPlace_1 is related to the actual object Saginaw of the original triple. This is done using properties which are of *statementProperty* and *valueProperty* and are constructed from the property birthProperty of original triple. Here five triples are required to depict a fact and any number of complex meta-information can be attached to the resource node.

**Table 3.** n-ary relations.

Subject	Predicate	Object
Serena Williams	birthplaceSub	birthplace_1
birthplace_1	birthPlaceVal	Saginaw
birthplaceSub	statementProperty	birthPlace
birthPlaceVal	valueProperty	birthPlace
birthplace_1	rdf:type	rdf: Statement
birthplace_1	dc:source	HistoryOfTennis

### RDF*

Another data model RDF* ^11^ was introduced by Hartig in 2017. Unlike earlier approaches, which introduced metadata as triples, this data model follows embedded triple. The fact triple is enclosed within double brackets and is assigned as the subject or object of the triple as shown in Table 4. In the table, the embedded triple, <<s,p,o>> refers to <<Serena Williams, birthplace, Saginaw>>. Any number of metadata can be added as the predicate and object of the triple. RDF* is extended to have nested triples.

**Table 4.** RDF*.

Subject	Predicate	Object
<< s, p, o>>	dc:source	HistoryOfTennis

RDF* data model is much more compact than other reification approaches and does not introduce any extra predicates like in singleton properties. It is also backward compatible with the RDF data model and other reification approaches. Although retrieving queries are simple compared to other approaches, various groups of annotations of the same fact (for example, the same incident happened at two different points in time) cannot be interpreted correctly. Frey et al. provide an alternative to overcome this by adding resource nodes for each annotation group. ^12^

## Methods

The trustworthiness of a KG is categorized into knowledge graph level, statement level and whether the statement indicates it does not have value or the value is not known. ^4^ In our research, we focus on trustworthiness defined at the statement level. Wikidata and YAGO provide statement-level provenance and contextual data, which is the focus of this paper and hence further explored in this section.

### Wikidata

Wikidata is one of the largest KG in machine-readable format on the Web. This curated and crowd-sourced dataset has a rich set of entity information. Wikidata uses its own data model and data is inherently stored in JavaScript Object Notation (JSON) format in the BlazeGraph database. But in order to effortlessly query Wikidata like other KGs, Wikidata provides RDF dumps and has introduced Wikidata SPARQL query service for querying Wikidata in RDF form. A large number of facts in Wikidata are annotated with metadata and therefore, widely uses reification to describe the facts. ^2^

Wikidata encodes facts in the form of item, label, description, alias and statement. Item describe an entity and different pieces of information about the entities are stored as primary relations (property-value pairs) of statements. This approach is very similar to the concept of representing facts as triple in RDF. Metadata of these facts are added by annotating statements with qualifiers, references and ranks.

Wikidata employs n-ary relations to cater for RDF annotations. ^6, 7^ Each entity is linked to the statement node and the statement node is linked to the value nodes. Any number of references, contextualization data and ranks can be added to statement nodes. For applications that do not require the complexity of accessing these n-ary relations, data in plain RDF without annotations is provided by Wikidata. A simplified relation between subject and object is provided as RDF triples for top rank statements.

The most popular qualifier for describing contextual data is temporal data.

**Figure 2.**
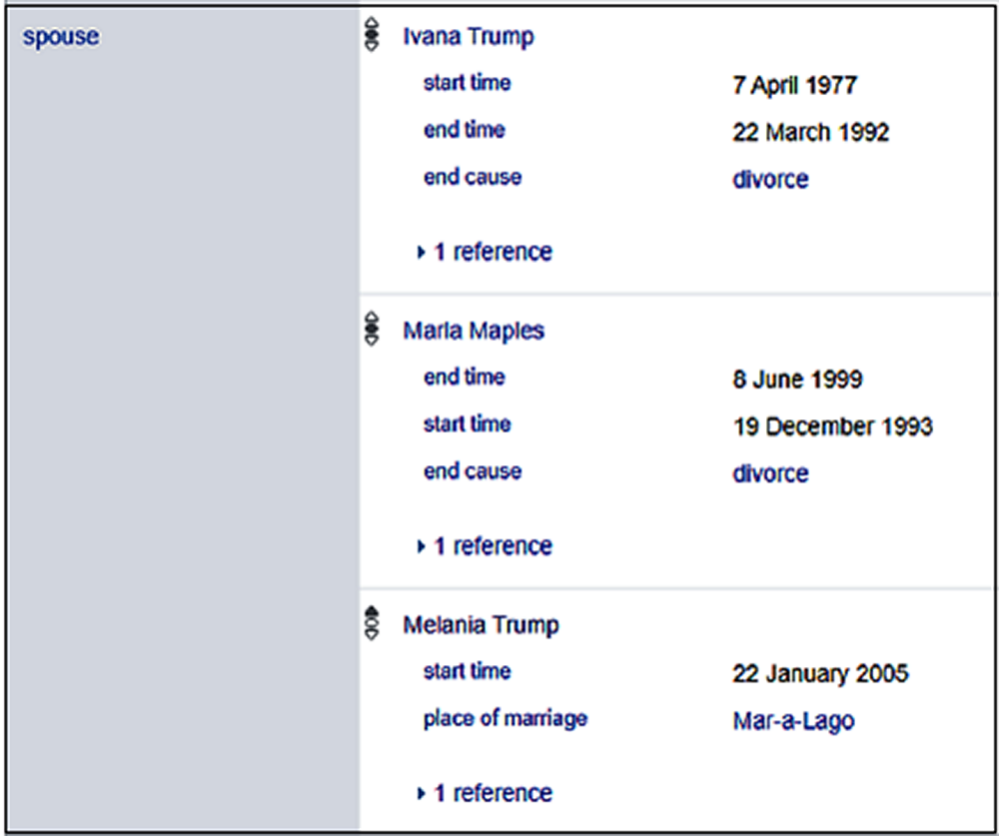
Qualifiers of statements of Donald Trump in Wikidata.

Wikidata resolves the awkward interpretation of many wives in Figure 1 by attaching temporal information to supplement the facts as displayed in Figure 2.

Wikidata allows property-value pairs to represent two special cases, one is if a property has a value, which is unknown and another is if a property does not have a value. *Some* is employed to represent an unknown value and *none* is employed to represent no value. This increases the trustworthiness of Wikidata.

**Figure 3.**
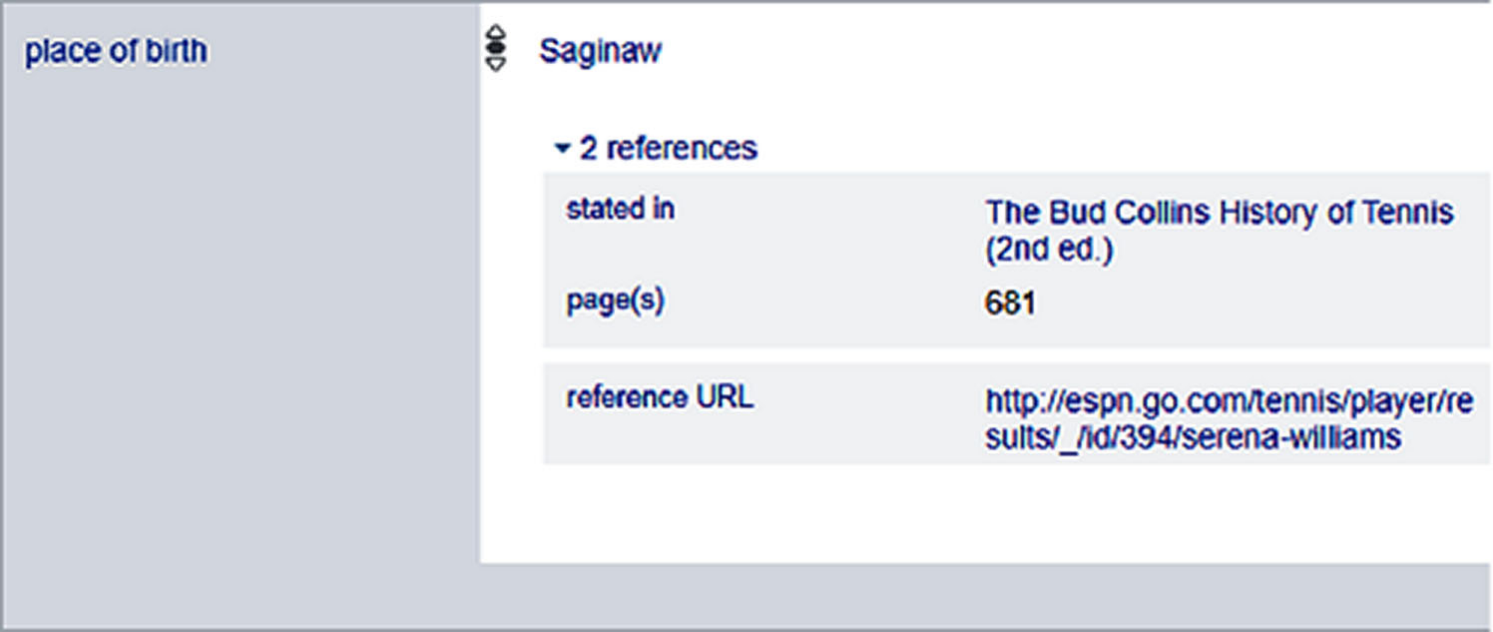
References of entity Serena Williams in Wikidata.

The source of the statements is referred by using predicates reference URL (P854), imported from (P143), stated in (P248), title (P1476) or publisher (P123). Wikidata references are optional and may be Wikidata items, links to websites, classical citations or datasets. More than one references are possible as depicted in Figure 3, where the references of the birthplace of American female tennis player Serena Williams is provided and serves as the provenance information.

If there are many statements involved for the same relations Wikidata provides ranks for filtering the result. *Preferred*, *normal* and *deprecated* are provided as ranks in the descending order of precedence.

**Figure 4.**
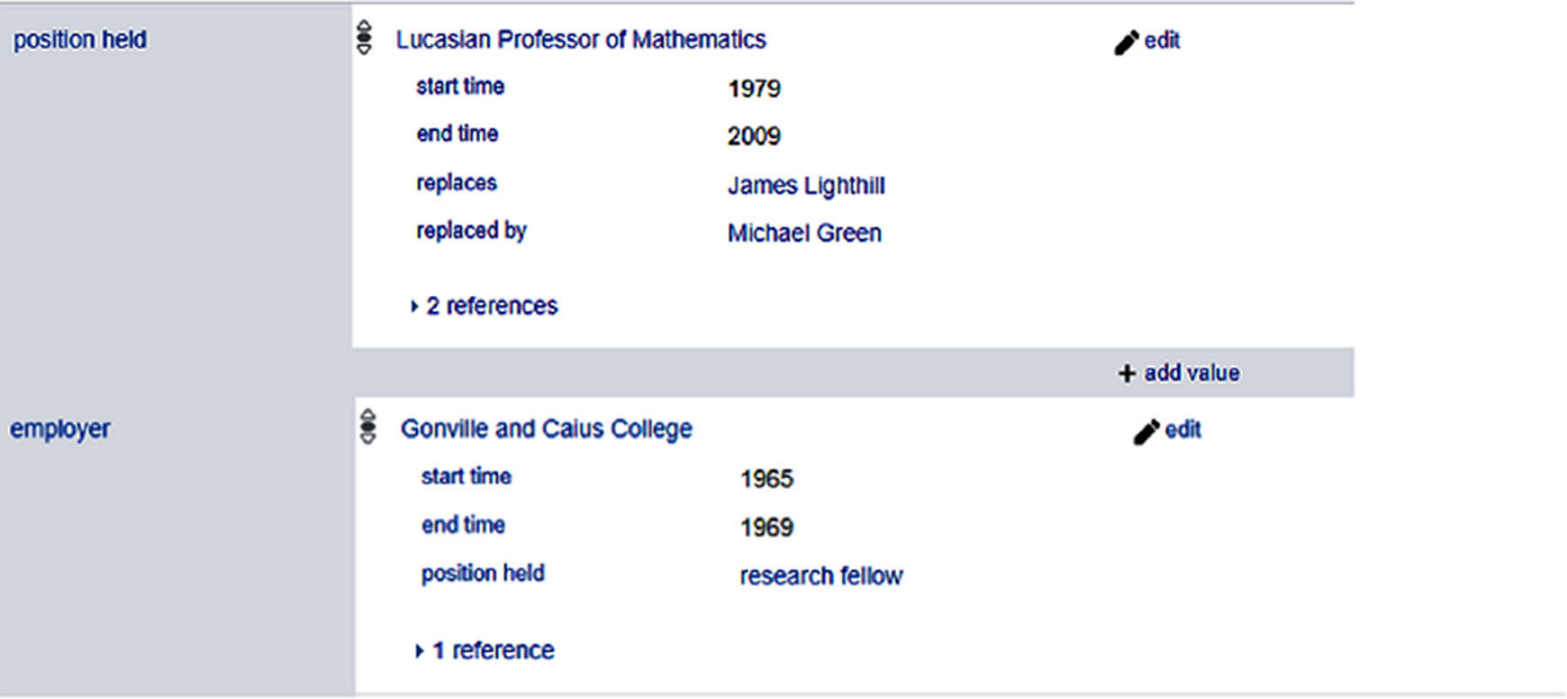
Excerpts of the position held by Stephen Hawking in Wikidata.

Wikidata uses the same predicate/property as part of primary relations and qualifiers. In Figure 4, the same predicate position held (P39) is used in primary relation (Stephen Hawking, position held (P39), Lucasian Professor of Mathematics) and as contextual information of the fact (Stephen Hawking, employer (P108), Gonville and Caius College). As we can see, here there is an inconsistency as to how to include the information of position held in a workplace.

Wikidata has more than a hundred and fifty qualifiers. Being a dynamic KG, qualifiers can keep getting added to the KG. Therefore Patel-Schneider ^13^ states that if the number of qualifiers attached to a fact is more, it is not trivial work to infer the trustworthiness of a fact. Therefore, limiting the context information to specific needs might be the smarter way for balancing the complexity of inferring trustworthiness.

### YAGO

YAGO4 employs RDF* data model to annotate facts in RDF format. YAGO4 is a combination of rich instance data from Wikidata and the ontology from schema.org. YAGO is constructed with very stringent measures to control noise. Facts which does not abide by these strict constraints are rejected. Semantic constraints like disjointness, domain and range restrictions, functional constraints on relations, cardinality constraint of objects are enforced on the dataset and has an accuracy of 95%. For the case in Figure 1, YAGO4 keeps only statements with the best rank from Wikidata, thus avoiding stale facts in KG.

YAGO4 provides temporal information to the facts by employing nesting of triples using RDF* data model. RDF* provides SPARQL* for easy accessing data on the Web. YAGO4 provides temporal dimension using two general predicates *startDate* and *endDate*, which can be applied to any class.

The previous version, YAGO2, ^14, 15^ built from Wikipedia, GeoNames and WordNet is furnished with more contextualization and provenance data. YAGO2 employed a tuple type, sextuples, which is called SPOTLX. SPOTLX representation uses six tuples, with time, location and context added to the triple (s, p, o), which is of the form (Subject, Predicate, Object, Time, Location, conteXt).

## Discussion

Based on the investigation, Wikidata has better coverage of contextualization and provenance data of real-world entities. For applications which is less susceptible to noise and more sensitive to specific contexts, the role of this meta information is really tremendous. YAGO4 provides coverage for temporal information. YAGO2 is more enriched with spatial and temporal facts and is provided with many predicates for including meta information.

The discussion of metadata in KGs is not complete without mentioning the downside. These provenance and contextual data come with a cost. The size of the KGs, which are already in humongous size, will further increase with the addition of metadata. Besides, the size depends on the approach used by each KG and the type and the number of provenance and contextualization data employed. The complexity of data retrieval is also another issue to be considered.

In order to reduce the effect of these downsides, the data in KGs are provided without annotations too, that is without employing reification. Both Wikidata and YAGO4 are available in RDF format, without metadata information.

In another angle, for applications where provenance data is crucial, all facts in KGs are not furnished with annotations. Nevertheless, this issue is under constant improvement.

The investigation outcome paves the way for further research to apply provenance and contextualization information for identifying fully reliable and truthful facts from knowledge graphs.

## Conclusions

In this paper, we have discussed RDF reification methods for annotating knowledge graphs with metadata. Two prominent knowledge graphs, namely Wikidata and YAGO4 were investigated. The method of incorporating metadata by these KGs and the extent of contextualization and provenance data supported by them are analyzed. Wikidata is equipped with higher level of contextualization and provenance data whereas YAGO4 is equipped with temporal information. For applications which do not require metadata, both Wikidata and YAGO are available in RDF format too, and thus reducing the complexity of the addition of metainformation. The findings obtained will be further incorporated for the detection of errors in KG, in order to increase their trustworthiness.

## Data availability

The data are taken from Wikidata and Yago.

## Author contributions

Sini Govndapillai did the conception of the work, data collection, data analysis and interpretation, drafting the article, and revision to the final version under the guidance of her supervisors, Lay-Ki Soon and Su-Cheng Haw. Su-Cheng Haw is the corresponding author for this paper.
