# Supplementary material for: An empirical study on Resource Description Framework reification for trustworthiness in knowledge graphs
Source: F1000Res. 2021 Nov 29;10:881. Originally published 2021 Sep 2. [Version 2] doi: 10.12688/f1000research.72843.2 (PMC8634049; doi:10.12688/f1000research.72843.2)
Supplement: Peer review history

# Peer review: Reviewer response for version 2

**DOI:** 10.5256/f1000research.79774.r101427

**Published:** 2021-12-01

**Authors:** Kok-Chin Khor

**Affiliations:** 1 Lee Kong Chian Faculty of Engineering Science, Tunku Abdul Rahman University, Kajang, Malaysia

I have no other comments.

Is the work clearly and accurately presented and does it cite the current literature?

Partly

If applicable, is the statistical analysis and its interpretation appropriate?

Not applicable

Are all the source data underlying the results available to ensure full reproducibility?

No source data required

Is the study design appropriate and is the work technically sound?

Yes

Are the conclusions drawn adequately supported by the results?

Partly

Are sufficient details of methods and analysis provided to allow replication by others?

Yes

Reviewer Expertise:

Data Mining, Computer Networks.

I confirm that I have read this submission and believe that I have an appropriate level of expertise to confirm that it is of an acceptable scientific standard.

# Peer review: Reviewer response for version 2

**DOI:** 10.5256/f1000research.79774.r101428

**Published:** 2021-11-30

**Authors:** Weiguo Zheng

**Affiliations:** 1 Fudan University, Shanghai, China

The paper introduces the RDF reification methods. I have no more comments.

Is the work clearly and accurately presented and does it cite the current literature?

Partly

If applicable, is the statistical analysis and its interpretation appropriate?

Not applicable

Are all the source data underlying the results available to ensure full reproducibility?

No source data required

Is the study design appropriate and is the work technically sound?

Partly

Are the conclusions drawn adequately supported by the results?

Partly

Are sufficient details of methods and analysis provided to allow replication by others?

Yes

Reviewer Expertise:

knowledge graph

I confirm that I have read this submission and believe that I have an appropriate level of expertise to confirm that it is of an acceptable scientific standard.

# Peer review: Reviewer response for version 1

**DOI:** 10.5256/f1000research.76450.r95305

**Published:** 2021-10-25

**Authors:** Weiguo Zheng

**Affiliations:** 1 Fudan University, Shanghai, China

**General Comments:**

 This paper focuses on RDF reification and introduces several approaches with examples. Then the author investigated the reification methods used by Wikidata and YAGO. However, there is no new method proposed in this paper, and the reification model, the query method and the ranking method are not well organized when analysing Wikidata and YAGO.

**Strong Points:**

1. There are enough examples to make relevant approaches easy to understand. The examples of Donald Trump and Serena Williams are throughout the paper to make relevant approaches easy to understand.
2. Several different methods from 2004 to 2017 are covered.

**Weak Points:**

1. The structure of the abstract is not clear. For example, the analysis of YAGO4 and Wikidata is interspersed in the utility of provenance data.
2. A specific definition of KG trustworthiness and RDF reification are needed.
3. Some applications of trustworthiness need to be introduced to make RDF reification more meaningful.
4. No new method has been proposed in this paper. In the Methods section, the authors only analyse the methods described in the Introduction section used by Wikidata and YAGO.
5. In the sections of Wikidata and YAGO, the contents of the reification method, the query method and the ranking method are organized without clear structures.

**Detailed Comments:**

1. In the abstract, “Therefore, the provenance of knowledge can assist in building up the trust of these knowledge graphs.” and “Therefore, the reliability of data in knowledge graphs can be increased by provenance data.” are repetitive.
2. In the Introduction section, “These facts can be classified as the provenance of knowledge and can contribute to the trust of these KGs.” is confusing due to the phrase of “facts”. In other parts of this paper the word “fact” refers to a triple in the KG.
3.  “Reification approaches” needs to be a section with the same level as “Introduction” and “Methods”.
4. In Wikidata section, “Wikidata uses the same predicate as part of primary relations and qualifiers.” needs more explanation to clarify the difference between predicate and primary relation.
5. The results from the Discussion section can be added to the Conclusions section.

Is the work clearly and accurately presented and does it cite the current literature?

Partly

If applicable, is the statistical analysis and its interpretation appropriate?

Not applicable

Are all the source data underlying the results available to ensure full reproducibility?

No source data required

Is the study design appropriate and is the work technically sound?

Partly

Are the conclusions drawn adequately supported by the results?

Partly

Are sufficient details of methods and analysis provided to allow replication by others?

Yes

Reviewer Expertise:

knowledge graphs

I confirm that I have read this submission and believe that I have an appropriate level of expertise to state that I do not consider it to be of an acceptable scientific standard, for reasons outlined above.

# Peer review: Reviewer response for version 1

**DOI:** 10.5256/f1000research.76450.r93428

**Published:** 2021-09-06

**Authors:** Kok-Chin Khor

**Affiliations:** 1 Lee Kong Chian Faculty of Engineering Science, Tunku Abdul Rahman University, Kajang, Malaysia

This paper provides an analysis on Wikidata and Yago4 related to provenance data and contextualisation. My comments are as follows:

- The finding of comparing Wikidata and Yago4 can be stated in the Abstract section.
- A more detailed explanation on RDF is needed to help the readers to understand the semantic web representation. Please refer to [3].
- The content in the Methods section is more towards analysis.
- The Conclusion section, besides restating the research method and summarise your overall arguments, you may want to summarise your finding and reiterates the important parts for supporting your claim.
-  An error in Page 4, second paragraph:
  "Additionally, some of the popular reification approaches standard reification, singleton property..."

Is the work clearly and accurately presented and does it cite the current literature?

Partly

If applicable, is the statistical analysis and its interpretation appropriate?

Not applicable

Are all the source data underlying the results available to ensure full reproducibility?

No source data required

Is the study design appropriate and is the work technically sound?

Yes

Are the conclusions drawn adequately supported by the results?

Partly

Are sufficient details of methods and analysis provided to allow replication by others?

Yes

Reviewer Expertise:

Data Mining, Computer Networks.

I confirm that I have read this submission and believe that I have an appropriate level of expertise to confirm that it is of an acceptable scientific standard.
